# Determination of the 95% effective dose of remimazolam tosylate in anesthesia induction inhibits endotracheal intubation response in senile patients

**DOI:** 10.3389/fphar.2023.1136003

**Published:** 2023-05-30

**Authors:** Liangchao Qu, Mei Liu, Ru Ouyang, Tianyuan Li, Dingde Long, Yao Jiang, Chengyu Wang, Liqin Cheng

**Affiliations:** ^1^ Department of Anesthesiology, The First Affiliated Hospital of Nanchang University, Nanchang, Jiangxi, China; ^2^ Department of Anesthesiology, Shanghai Jiao Tong University Affiliated Sixth People’s Hospital, Shanghai, China

**Keywords:** biased coin design, benzodiazepines, anesthesia, intubation, aged

## Abstract

**Background and Purpose:** The prevalence of elderly patients prompts anesthesiologists to determine the optimal dose of medication due to the altered pharmacokinetics and pharmacodynamics of this population. The present study aimed to determine the 95% effective dose (ED_95_) of remimazolam tosylate in anesthesia induction to inhibit endotracheal intubation-related cardiovascular reaction in frail and non-frail senile patients.

**Methods:** A prospective sequential allocation dose-finding study of remimazolam tosylate was conducted on 80 elderly patients who received general anesthesia between May and June 2022 at the First Affiliated Hospital of Nanchang University. The initial dose was 0.3 mg/kg. The blood pressure and heart rate fluctuations during intubation were either <20% (negative cardiovascular response) or ≥20% (positive cardiovascular response). If positive, the dose of the next patient was increased by 0.02 mg/kg, while if negative, it was reduced by 0.02 mg/kg according to the 95:5 biased coin design (BCD). The ED_95_ and 95% confidence intervals (CIs) were determined using R-Foundation isotonic regression and bootstrapping methods.

**Results:** The ED_95_ of remimazolam tosylate to inhibit the response during tracheal intubation was 0.297 mg/kg (95% CI: 0.231–0.451 mg/kg) and 0.331 mg/kg (95% CI: 0.272–0.472 mg/kg) in frail and non-frail senile patients, respectively.

**Conculation and Implications:** The CI of the two groups overlap, and no difference was detected in the ED_95_ of remimazolam tosylate in inhibiting endotracheal intubation-related cardiovascular response in frail and non-frail senile patients. These results suggested that remimazolam tosylate is an optimal anesthesia inducer for all elderly patients.

**Clinical Trial Registration:** https://www.chictr.org.cn, identifier ChiCTR2200055709.

## Introduction

The operation rate of elderly patients is rising continually, and the proportion of elderly patients aged >65 years undergoing the operation is about 37% (Hall et al., 2010). In addition, >30% of elderly patients also present weakness before surgery and anesthesia (McIsaac et al., 2020a). Previous studies have shown that compared to the non-frail population; frail elderly have a higher incidence of postoperative complications and increased mortality (Chan et al., 2021; Kikuchi et al., 2021). Therefore, perioperative management of elderly patients, especially weak individuals, poses significant challenges for anesthesiologists.

Tracheal intubation stimulation under general anesthesia activates the sympathy-adrenal medullary and renin-angiotensin systems, which significantly increases the release of catecholamines, causing tachycardia hypertension, arrhythmia, and myocardial ischemia in patients (Yang et al., 2020). Anesthesia induction and endotracheal intubation can cause significant fluctuations in hemodynamics in elderly patients (Jalali et al., 2017). Some studies have shown that compared to non-frail people; frail elderly have a lower stroke volume (Gharacholou et al., 2015) and a diminished heart rate (HR) response (Parvaneh et al., 2015). Thus, frail patients are more likely to suffer hemodynamic fluctuation during anesthesia induction and tracheal intubation than non-frail patients. The hemodynamic instability caused by anesthesia-inducing drugs and the sharp hemodynamic change during tracheal intubation increases the perioperative cardiovascular accident (Licker et al., 1995) and stroke risk. Therefore, maintaining hemodynamic stability during anesthesia induction and endotracheal intubation in elderly patients is imperative.

Although several methods have been attempted to prevent hemodynamic changes in patients during intubation (Talebi et al., 2010; Teong et al., 2020; Hajian et al., 2021), none of them are perfect and pose a few complications. Remimazolam tosylate has the characteristics of rapid onset, short maintenance and recovery time, no accumulation, and metabolism independent of liver and kidney function (Borkett et al., 2015; Rex et al., 2021). Compared to propofol, remimazolam tosylate exerts equivalent sedation, has more stable hemodynamics (Chen et al., 2020; Doi et al., 2020; Chen et al., 2021), and is safe and effective in high-risk ASA patients (Rex et al., 2021; Shi et al., 2022), rendering it suitable for general anesthesia in elderly patients (Nakayama et al., 2021). Thus, remimazolam tosylate can be safely and effectively used for anesthesia induction (Liu et al., 2021a; Nakanishi et al., 2021). This makes it an ideal anesthesia induction drug for elderly patients (Nakanishi et al., 2021; Oka et al., 2021). Dai et al. showed that the sedation success rates of 0.2, 0.3, and 0.4 mg/kg of remimazolam tosylate were 89%, 94%, and 100%, respectively (Dai et al., 2021). Sun et al. demonstrated that the 95% effective dose for successful gastrointestinal sedation in elderly patients was 0.162 mg/kg [95% confidence interval (CI): 0.160–0.166 mg/kg] (Sun et al., 2022). During cardiac surgery, anesthesia-induced side effects were significantly reduced by 10.00% and 30.00% with 0.3 mg/kg remimazolam compared to 1.5 mg/kg propofol, respectively (*p* < 0.05) (Tang et al., 2021). However, the optimal dose for endotracheal intubation is yet uncertain in elderly patients.

Therefore, the present study aimed to explore the 95% effective dose (ED_95_) of remimazolam tosylate anesthesia to inhibit tracheal intubation reaction in senile patients with and without weakness and provide a reference for clinical medication.

## Methods

### Study design and subject selection

This prospective, sequential allocation, dose-finding trial recruited 80 candidates aged >65 years between May 2022 and June 2022 in the First Affiliated Hospital of Nanchang University in Nanchang, China. The study was registered with the China Clinical Trials Center (ChiCTR2200055709) and approved by the Ethics Committee of the First Affiliated Hospital of Nanchang University (AF-SG-03-2.0). Written informed consent was obtained from the patients or authorized individuals.

The inclusion criteria were as follows: 1) Patients must be > 65-years-old and candidates for elective surgery; 2) Physical status of ASA I–III (Mayhew et al., 2019); 3) Body mass index (BMI) 20–25 kg/m^2^; 4) Patients who intend to undergo single-lumen endotracheal tube surgery under general anesthesia; 5) Informed consent form signed by the patient or authorized person. The exclusion criteria were as follows: 1) First intubation failed or the patient withdrew from the trial; 2) Patient’s alertness/sedation score was more significant than 1 point post-remimazolam tosylate injection after 3 min; 3) Patients intermittently used benzodiazepines shortly before the surgery; 4) Patients who had a history of hypertension and baseline systolic blood pressure (SBP) > 180 mmHg; 5) Patients with severe obstructive (chronic obstructive pulmonary disease gold ≥3) or restrictive (forced vital capacity <80% of predicted value) pulmonary disease or ischemic heart disease; 6) Test drug allergy; 7) Nervous system disease or inability to communicate appropriately.

### Study protocol

The patient’s preoperative fasting status was confirmed in compliance with ASA guidelines. No premedication was used. An intravenous (i.v.) cannula was inserted in the pre-anesthesia room, and 6–8 mL/kg of lactated Ringer’s solution was infused. Radial artery puncture and catheterization were performed under local anesthesia by an anesthetist. Standard monitoring with continuous electrocardiography, invasive arterial BP monitoring, and pulse oximetry (S_p_O_2_) was used for frail and non-frail groups during the trial.

Elderly patients >65-years-old were assessed for weakness on the clinical frailty scale (CFS) (McIsaac et al., 2020b) the day before the operation. The CFS is a 9-point global rating scale; the score is assigned based on the assessment of mobility, energy levels, physical activity, and function. The primary study physician conducted all frailty assessments. Subjects with CFS scores <4 were included in the non-frail group, and those with scores ≥4 comprised the frail group.

Before administration, 2 mg/mL remimazolam tosylate (Jiangsu Hengrui Pharmaceutical Co., Ltd, Jiangsu, China) was prepared by diluting 36 mg remimazolam tosylate in 0.9% saline (18 mL). The study dose (6.63–12.48 mL) was further diluted in saline to a total volume of 15 mL. A bolus of targeting remimazolam tosylate was administered via an infusion pump (Shenzhen Mindray Bio-Medical Electronics Co., Ltd, Shenzhen, China) for 1 min, followed immediately by a flush of saline (5 mL). Dose sequencing was based on the biased coin design (BCD) method, with an initial dose of 0.3 mg/kg, and the dose gradient was 0.02 mg/kg for frail and non-frail groups. Dai et al. (Dai et al., 2021) showed a 94% success rate of anesthesia induction with 0.3 mg/kg remimazolam, deeming it a safe dose in clinical practice; hence, 0.3 mg/kg remimazolam was selected as the starting dose for both groups of patients in the trial. If the cardiovascular response to endotracheal intubation was positive in the previous patient, the next patient received an increasing dose gradient of remimazolam, while if the response was negative, the next patient had a 5% random probability of one dose gradient of remimazolam reduced and a 95% probability of the same dosage as the previous patient.

**FIGURE 1:**
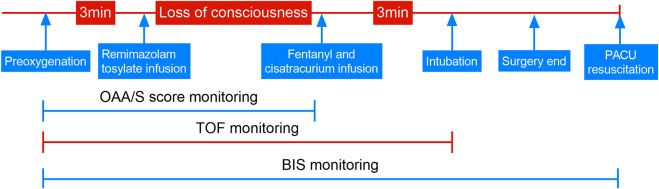
Trial sequence. OAA/S, observers’ assessment of alertness/sedation; TOF, train of four stimulations; BIS, bispectral index; PACU, post-anesthesia care unit.

Upon arrival in the operation room and after preoxygenation, anesthesia was induced by targeting remimazolam tosylate for sedation. When the observers’ assessment of alertness/sedation (OAA/S) score (Chernik et al., 1990) was ≤1 point (unresponsive to mild shaking or prodding) or the bispectral index (BIS VISTATM, GE Healthcare, United States) was ≤60 after the patient’s consciousness disappeared, sedation was considered successful. Then, 4 μg/kg fentanyl (Yichang Humanwell Pharmaceutical Co., Ltd, Yichang, China) and 0.15 mg cisatracurium (Hangzhou Hongyou Pharmaceutical Technology Co., Ltd, Hangzhou, China) were administered for complete anesthesia induction. An experienced anesthetist used a video laryngoscope to intubate the patient if there was no response to train-of-four (TOF) stimulation 3 min after the infusion of fentanyl and cisatracurium. SBP, diastolic arterial BP (DBP), and HR were recorded by another experienced anesthesiologist, blinded to the remimazolam dose, at the following time points: baseline vital signs before induction (T0); successful sedation (T1); immediately before intubation (T2); immediately after intubation (T3); 30 s, 1 min, and 2 min post-intubation (T4, T5, and T6, respectively), using sustainable monitoring. Subsequently, the expected operation was performed. At the end of the procedure, the subjects were admitted to the post-anesthesia care unit (PACU) for resuscitation, wherein all subjects were assessed for awareness by the anesthesia nurse. In this study, BIS monitored the depth of anesthesia, and TOF monitored the degree of muscle relaxation until the end of endotracheal intubation. The detailed sequence of this trial is shown in Figure 1.

If the absolute HR was <45 bpm or the baseline mean arterial pressure (MAP) was decreased by >30% (Wesselink et al., 2015), immediate treatment was given with 0.3 mg atropine or 4 µg norepinephrine. The symptomatic treatment can be reused if necessary. If the MAP or HR is higher than the baseline value by >30%, it should be carefully observed for 1 min. If there is no remission, anesthesia or urapidil 5 mg or esmolol 0.5 mg/kg is deepened. These phenomena were regarded as adverse complications and recorded in the current study.

The positive responses were defined as MAPmax or HRmax ≥20% of the fundamental value within 2 min after intubation. Negative responses were defined as either the variation of MAPmax and HRmax during intubation at < 20% of the baseline value. Failure to sedate with the target dose of remimazolam tosylate was also considered a positive reaction, and remimazolam tosylate was administered further to deepen anesthesia; subsequently, the patient was withdrawn from the trial.

### BCD method

The BCD method was used to determine the adequate dose level of remimazolam tosylate at Г = 0.95 quantiles for frail and non-frail senile patients. Г is the level of drug effect determined by the study purpose. Since ED95 is desired, Г = 0.95 is assigned to produce a response in 100×Г = 95% of the target population (Pace and Stylianou, 2007). The K-sequential dose level was selected to increase or decrease the dose by 0.02 mg/kg between classes, and consecutive patients were exposed to one of the successive K-dose levels with an initial dose of 0.3 mg/kg. The recommended dose for the current study (Dai et al., 2021) was implemented at investigator’s discretion. The research dose in the current study was 0.26, 0.28, 0.30, 0.32, and 0.34 mg/kg, respectively. If the cardiovascular response to endotracheal intubation was positive in the previous patient, the next patient received an increased dose gradient of remimazolam, while in the case of a negative response, the next patient had a 5% random probability of reduced remimazolam by one dose gradient and a 95% probability of maintaining the dose level same as the previous patient.

## Outcomes

The primary outcome was to determine the ED_95_ of remimazolam tosylate in anesthesia induction to inhibit endotracheal intubation reaction in frail and non-frail senile patients.

The secondary outcomes included HR and BP changes during tracheal intubation and adverse reactions, such as the amount of vasoactive drugs, sedation failure, hypotension (Wesselink et al., 2015) (baseline MAP reduction >30%), bradycardia (HR < 45 bpm), low SpO2, and injection pain.

### Sample size calculation

In the BCD study, *a priori* calculation of the sample size was not possible due to the non-independence (the dose administered to the next patient was dependent on the intubation response of the previous patient) and the unknown dose distribution. The parametric estimates of all target doses were balanced in approximately 20 subjects and stabilized in 40 subjects, as described previously (Stylianou and Flournoy, 2002). Therefore, 40 subjects were recruited for each group in this study.

### Statistical analysis

Isotonic regression with R Foundation was used to calculate the ED_95_ estimate of û3 (Pace and Stylianou, 2007), which is the linearly interpolated dose between P* κ and P* κ+1 at the probability of Г = 0.95. P* κand P* κ+1indicated adjusted response rates at doses χ _κ_ and χ _κ+1_, respectively. The adjusted response rate is calculated monotonically using the pool-adjacent-violators algorithm (PAVA), following which the response rate was recalculated by pooling the adjacent increasing and decreasing pairs. Isotonic regression is an adjustment of regression that limits the probability of a sequential dose response to be monotonic. The 95% CI for ED_95_ was calculated using a bias-corrected percentile derived by bootstrapping with a resampling size of 40, a repeat number of 2000, and a target Г of 0.95. The above data were analyzed using R Foundation.

SPSS 26.0 software (IBM SPSS Inc, Armonk, NY, United States of America) was used for data analysis. The mean [standard deviation (SD)] of measurement data of normal distribution was computed. The comparison between the two groups was performed using independent sample *t*-test. In the case of non-normal data distribution, median (interquartile range, IQRs) was used, and the comparison between the two groups was conducted using a non-parametric Mann–Whitney *U* test with two independent samples. The enumeration data were expressed as absolute numbers, and the comparisons between groups were performed using the χ^2^ test. Fisher’s exact probability test was applied where appropriate. Baseline BP and HR were compared between the two groups using unpaired Student’s t-test, and two-factor analysis of variance was used for repeated measures data, followed by Sidak’s test (*p* < 0.05) for multiple comparisons. *p* < 0.05 indicated a statistically significant difference.

## Results

### Baseline characteristics

**FIGURE 2:**
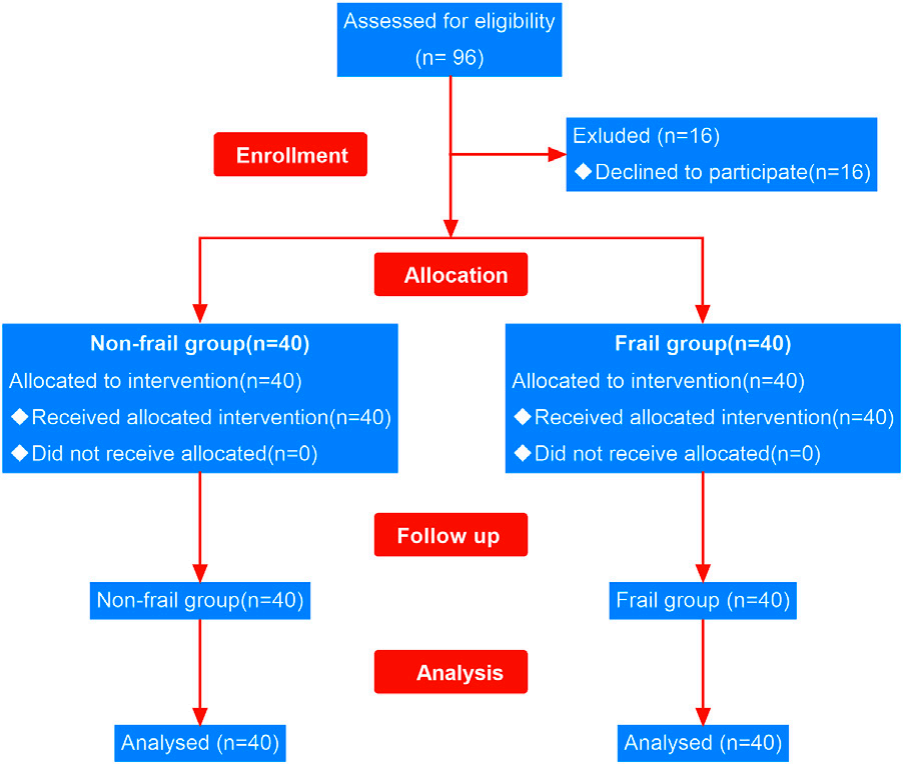
Flow chart of participants in the study.

A total of 96 patients undergoing elective surgery were recruited in this study, of which 16 declined the trial as their independent choice. The remaining 80 subjects (40 frail and 40 non-frail) completed the study (Figure 2). The analysis of demographic characteristics of the frail and non-frail groups did not show any significant differences in age, gender composition, BMI, and ASA classification (Table 1).

**TABLE 1:** Demographic characteristics.

Demographic and clinical features	Non-frail group (N = 40)	Frail group (N = 40)	*p*-value
Age (years), median (IQR)	67.5 (65.0–70.0)	69.0 (67.0–72.0)	0.178
Sex, n(%)			
Male sex	25(62.5)	20(50.0)	0.260
Female sex	15(37.5)	20(50.0)	
Weight (kg)	57.5(51.1-60.0)	58.2 (6.9)	0.563
Height (cm), mean(SD)	161.4 (6.3)	160.3 (6.9)	0.441
BMI (kg/m^2^)	22.2 (2.2)	22.3 (21.2-23.9)	0.411
CFS weak score, median (IQR)	3.0 (2.0–3.0)	7.0 (7.0–8.0)	<0.001
ASA physical status, n(%)			
Ⅱ	28(70.0)	22(55.0)	0.166
Ⅲ	12(30.0)	18(45.0)	

IQR, inter-quartile range; SD, standard deviation; BMI, body mass index; CFS, clinical frailty scale; ASA, American Society of Anesthesiologists.

### Dose-response

**FIGURE 3:**
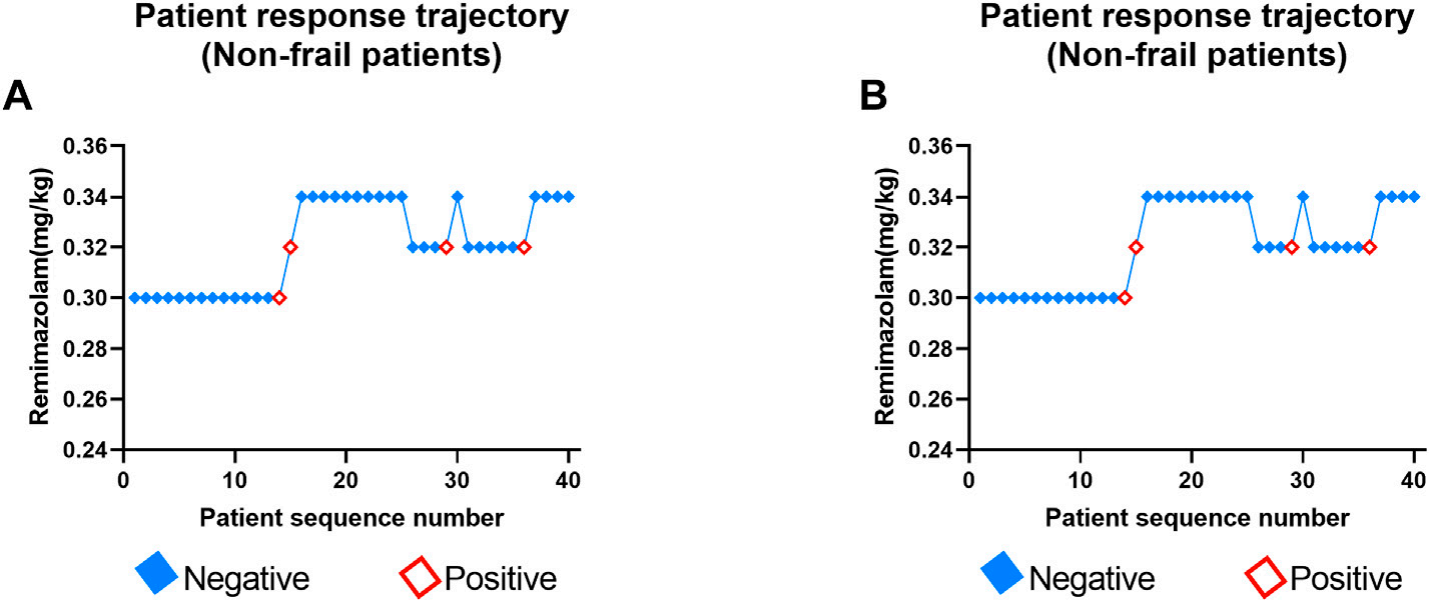
Determination of ED_95_ of remimazolam tosylate in anesthesia induction to inhibit endotracheal intubation reaction in non-frail **(A)** and frail groups **(B)**. Patient number (x-axis) is the exposure sequence of subjects with BCD. The specified dose (y-axis) of remimazolam tosylate was 0.26, 0.28, 0.30, 0.32, and 0.34 mg/kg, respectively. A solid square indicates a negative reaction to tracheal intubation; a hollow square indicates a positive reaction.

The positive or negative reaction of each subject to the specified dose of remimazolam tosylate in the two groups is shown in the standard plot, with the x-axis as the sequence of subjects and the y-axis as the dose of each subject (Figure 3). The specific response doses (y-axis) of remimazolam tosylate were 0.30, 0.32, and 0.34 mg/kg in the non-frail subjects compared to 0.26, 0.28, and 0.30 mg/kg in frail subjects, respectively. One candidate reacted positively at 0.30 mg/kg, and three candidates were positive to 0.32 mg/kg in the non-frail patient group (Figure 3A), while in the frail patient group, one was positive to 0.26 mg/kg and the other to 0.28 mg/kg dose (Figure 3B).

The number of subjects tested, the number of negative subjects, the observed response rates, and the PAVA-adjusted response rates for both groups at different target doses are presented in Table 2. In the non-frail group, the observed response rate was 0.927, 0.727, and 1.000 at the assigned doses of 0.30, 0.32, and 0.34 mg/kg of remimazolam tosylate, while the PAVA-adjusted response rate was 0.870, 0.920, and 1.000, respectively. The observed response rate decreased with increasing doses, but the response rate after PAVA adjustment did not decrease with increments in the dose. The estimated ED_95_ using isotonic regression was 0.331 mg/kg (95% CI: 0.272–0.472) in the non-frail group and 0.297 mg/kg (95% CI:0.231–0.451) in the frail group. Overlapping CIs did not show any significant difference in ED_95_ between the two groups.

**TABLE 2:** Observed and PAVA-adjusted response rates with remimazolam (isotonic regression method) in non-frail and frail senile patients.

Assigned dose(mg/kg)	Number negative	Number tested	Observed response rate	PAVA-adjusted response rates
Non-frail patients				
0.30	13	14	0.929	0.870
0.32	8	11	0.727	0.920
0.34	15	15	1.000	1.000
Frail patients				
0.26	2	3	0.667	0.630
0.28	7	8	0.875	0.940
0.30	29	29	1.000	1.000

### BP and HR fluctuation

**FIGURE 4:**
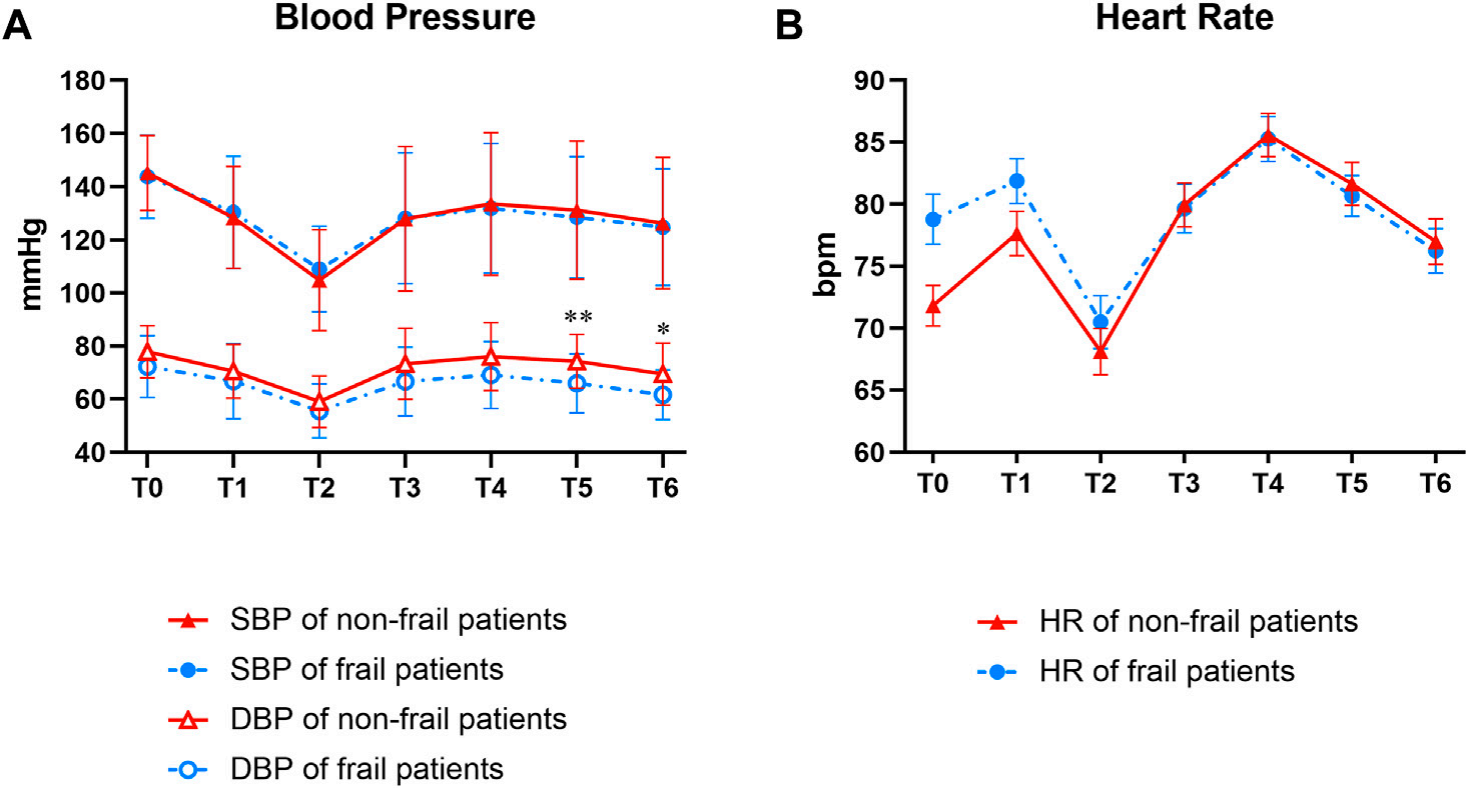
Groups comparison of data relevant to BP **(A)** and HR **(B)**. T0, T1, T2, T3, T4, T5, and T6 indicated time points before induction, successful sedation, immediately before intubation, immediately after intubation, and 30 s, 1 min, and 2 min post-intubation, respectively. At T5, and T6, the DBP is significantly higher in the non-frail group than in the frail group (**p = 0.006 and *p = 0.010, respectively). No significant difference was detected in SBP and in HR between the non-frail and frail groups. BP, blood pressure; DBP, diastolic BP; SBP, systolic BP; HR, heart rate.

DBP was significantly higher in the non-frail group than in the frail group at 1 and 2 min post-intubation (*p* = 0.006 and 0.010, respectively). However, no significant differences were detected in the SBP and HR between the two groups (Figure 4).

## Adverse events

Injection pain reaction was observed in two patients in the non-frail group but none in the frail group. The vital signs of all the patients were stable; hence, no vasoactive drugs were used. All patients were sedated successfully with the target dose of remimazolam tosylate, and there was no delay in awakening after the operation. None of the patients had hypotension, low SpO_2_, or bradycardia in both groups.

## Discussion and conclusions

The present study demonstrated that the ED_95_ of remimazolam tosylate anesthesia induction to inhibit the cardiovascular response of senile patients with and without frailty was 0.297 mg/kg (95% CI: 0.231–0.451 mg/kg) and 0.331 mg/kg (95% CI: 0.272–0.472 mg/kg), respectively. The overlapping CIs did not show any significant difference in ED_95_ between the two groups. These results suggested that remimazolam tosylate is an optimal anesthetic inducer for elderly patients, irrespective of their frailty.

The ED_95_ dose in this study was significantly higher than the 0.162 mg/kg estimated previously (Sun et al., 2022). This phenomenon could be attributed to the following reasons: (1) The study by Sun et al. estimated the ED_95_ of gastroscopy in the elderly, but our study focuses on the ED_95_ of inhibition of endotracheal intubation reaction. The stimulation intensity of endotracheal intubation is higher than gastroscopy; hence, the dose is elevated. (2) Sun et al. used the Probit method to estimate ED_95_ based on ED_50_, and the dose may be small (Pace and Stylianou, 2007). Other studies have shown a success rate of 96.52% with 0.15 mg/kg of remimazolam colonoscopy (Liu et al., 2021b), possibly due to a small sedation dose.

Our trial used the isotonic regression method to derive the dose-response curve of remimazolam tosylate and estimated ED_95_. Isotonic regression has statistical characteristics that measure the dose effect at any percentile and has the lowest bias and variation (Pace and Stylianou, 2007; Wu et al., 2021). This BCD approach avoids the drawback of estimating ED_95_ using unproven ED_50_ (Pace and Stylianou, 2007; Tang et al., 2020; Zhu et al., 2020; Ni et al., 2022) because the peak dose distribution of ED_50_ is similar to the average. Therefore, the BCD method combined with the estimation of isotonic regression is often used in anesthesia studies (Mittal et al., 2019; Kewlani et al., 2021; Wu et al., 2021).

The hemodynamic changes caused by the body’s stress response can peak within 1 min after stimulation (Chen et al., 2015). The intubation process starts from laryngoscopic exposure to the end of endotracheal cannula inflation, which must be completed within 30 s. This test judges the tracheal intubation response based on the changes in MAP and HR within 2 min after the completion of intubation.

The present study showed that the arterial DBP of the non-frail group was significantly higher than that of the frail group at 1 and 2 min post-intubation, which might be because the vascular elasticity in the non-frail group is better than that in the frail patients. Previous studies have shown that frail elderly have lower stroke volumes and a diminished HR response compared to non-frail people (Gharacholou et al., 2015; Parvaneh et al., 2015). However, no significant difference was observed in SBP and HR between the two groups in this study, which might be since general anesthesia with remimazolam can maintain the balance between sympathetic and parasympathetic nerve activities (Hasegawa et al., 2022) without affecting the cardiac output and evident cardiac suppression (Furuta et al., 2021). Furthermore, the injection pain of remimazolam tosylate was 2.5%, consistent with 2.4% in Zhang’s trial (Zhang et al., 2021).

Subsequently, we observed that about 1 min after the injection of a given dose of remimazolam tosylate, the sedation of eyelash reflex, physical movement response, sedation score, and vital signs were successful, while the BIS value was >60 in the patients. The study by Shirozu also monitored this phenomenon (Shirozu et al., 2022) and found that the pupillary diameter is a parameter in assessing the sedation level during remimazolam anesthesia. In future research, higher quality literature is required to demonstrate whether pupil diameter monitoring the depth of anesthesia is superior to BIS in remimazolam tosylate anesthesia.

### Study limitations

The present study has several limitations. First, we did not draw blood to detect changes in the blood stress indicators during endotracheal intubation. Second, the fluctuation in BP and HR within 20% in the intubation reaction was defined as successful inhibition of intubation. Although there was only a slight fluctuation, it may eventually lead to a large ED_95_. Finally, only BP and HR changes during endotracheal intubation were monitored, and no cardiac output changes were observed using cardiac function monitoring.

## Data Availability

The original contributions presented in the study are included in the article/supplementary materials, further inquiries can be directed to the corresponding authors.
